# P343: Management of hospital risks : between emergency and care quality in Algeria

**DOI:** 10.1186/2047-2994-2-S1-P343

**Published:** 2013-06-20

**Authors:** D Zoughailech

**Affiliations:** 1 Department of medicine preventive and épidemiology , University Hospital of Constantine, Constantine, Algeria

## Introduction

The stake represented by risks from care environment is not well perceived in Algeria. The absence of a regulatory framework; insufficiency of surveillance, prevention and control of healthcare-associated infections and the mismanagement of waste arising from care activities involving infectious risks are few illustrations about it.

## Objectives

Establishing a diagnosis of the current risks in our hospitals and show the urgency of their care, Propose a quality approaches for risk reduction.

## Methods

Our approach consists in analyzing surveys (Prevalence of healthcare associated infections and surgical-site infections, SSI), the practices audit and the reports made by our hospital hygiene unit during the two years 2011/2012 in Universitary hospital of Constantine.

## Results

In terms of risk and quality of care, the nosocomial infection (NI) is currently one of the best "markers". According to our studies, the prevalence of NI was 14% and that of SSI 9%. We will record the endemicity of MRB (multi-resistant bacteria) and the emergence of vancomycin-resistant enterococci (VRE). We will discuss of the other insufficiencies, such as the behavior of health care professionals, about the very low adhesion rate to hand hygiene.

## Conclusion

Finally, we will propose a quality system oriented on responsibility shared by managers, professionals, technical services staff and patients. This approach is subject to a structured program to protect patients, improve the working conditions of staff and reduce risks.

## Disclosure of interest

None declared.

